# Supplementary material for: Causes of bullying among young people and protective mechanisms and preventative factors that promote mental health and well-being: young people’s perspectives
Source: Int J Qual Stud Health Well-being. 2025 Jun 30;20(1):2524459. doi: 10.1080/17482631.2025.2524459 (PMC12210411; doi:10.1080/17482631.2025.2524459)
Supplement: Main doc with authors information 15 June 20253b.docx [file ZQHW_A_2524459_SM2615.docx]

**Causes of bullying among young people and protective mechanisms and preventative factors that promote mental health and well-being: young people’s perspectives**

**Miia Hästbacka****^1^, Søren Harnow Klausen^2^, Heléne Dahlqvist****^3^, Yulia Korzhina^4^, Amanda J. Sundqvist^5^, Emelie Käcko^6^, Pia Nyman-Kurkiala^7^, Joakim Strindberg^8^, Jessica Hemberg^9*^**

1. *Department of Health Sciences, Faculty of Education and Welfare Studies, Åbo Akademi University, Vaasa, Finland. E-mail: [miia.hastbacka@abo.fi](mailto:miia.hastbacka@abo.fi). ORCID ID: <https://orcid.org/0009-0004-2270-7338>
2. Department of Design, Media and Educational Science, University of Southern Denmark, Odense, Denmark. E-mail: [harnow@sdu.dk](mailto:harnow@sdu.dk) ORCID ID: <https://orcid.org/0000-0002-2650-1861>
3. Department of Health Sciences, Mid Sweden University, Sundsvall, Sweden. E-mail: [helene.dahlqvist@miun.se](mailto:helene.dahlqvist@miun.se) ORCID ID: <https://orcid.org/0000-0003-3209-186X>
4. Department of Health Sciences, Faculty of Education and Welfare Studies, Åbo Akademi University, Vaasa, Finland. E-mail: [yulia.korzhina@abo.fi](mailto:yulia.korzhina@abo.fi) ORCID ID: <https://orcid.org/0000-0001-9307-7637>
5. Department of Health Sciences, Faculty of Education and Welfare Studies, Åbo Akademi University, Vaasa, Finland. E-mail: [amanda.sundqvist@abo.fi](mailto:amanda.sundqvist@abo.fi) ORCID ID: <https://orcid.org/0000-0001-7204-4895>
6. Department of Health Sciences, Faculty of Education and Welfare Studies, Åbo Akademi University, Vaasa, Finland. E-mail: [emelie.kacko@abo.fi](mailto:emelie.kacko@abo.fi) ORCID ID: https://orcid.org/0000-0001-7554-1125
7. Department of Social Sciences, Youth Science, Åbo Akademi University, Vaasa, Finland. E-mail: [pia.nyman-kurkiala@abo.fi](mailto:pia.nyman-kurkiala@abo.fi) ORCID ID: <https://orcid.org/0000-0002-1462-9251>
8. Department of Education, Faculty of Social Sciences, Linnӕus University, Kalmar, Sweden. E-mail: [joakim.strindberg@lnu.se](mailto:joakim.strindberg@lnu.se) ORCID ID: <https://orcid.org/0000-0001-6098-8389>
9. Department of Health Sciences, Faculty of Education and Welfare Studies, Åbo Akademi University, Vaasa, Finland. E-mail: [jessica.hemberg@abo.fi](mailto:jessica.hemberg@abo.fi) ORCID ID: <https://orcid.org/0000-0002-0829-8249>

Corresponding author’s details and e-mail address:

Department of Health Sciences, Faculty of Education and Welfare Studies, Åbo Akademi University, Strandgatan 2, 65100, Vaasa, Finland. E-mail: [miia.hastbacka@abo.fi](mailto:miia.hastbacka@abo.fi). ORCID ID: <https://orcid.org/0009-0004-2270-7338>

**Abstract**

**Purpose:** Several factors influence young people’s mental health, and exposure to bullying is a risk factor. Bullying and cyberbullying are strongly associated with loneliness, but little research has been conducted on this topic. This study aimed to explore the causes of bullying among young people and the protective mechanisms and preventive work that can promote well-being, as seen from young people’s perspectives.

**Methods:** A qualitative exploratory design and content analysis were conducted. The interviews were conducted with 35 young people (aged 17–28 years).

**Results:** Three main categories were identified: (1) causes of bullying, intervention, and mitigation; (2) protection against the harmful effects of bullying and loneliness; and (3) prevention and promotion of well-being as solutions. Nine subcategories were identified in this study.

**Discussion:** Further research could explore interventions to prevent bullying in school settings, focusing on how bullying prevention programs are implemented and how school staff and guardians perceive and work with bullying issues.

*Keywords:* bullying, mental health, loneliness, adolescents, young adults

**Introduction**

According to the World Health Organization (WHO, 2024a), many young people globally suffer from mental health problems. During adolescence, mental health is influenced by several factors, of which peer relationships are important and bullying is considered a risk factor (World Health Organization, 2024a). Adolescents with long-term mental health problems are more likely to experience traditional and cyberbullying (Fridh et al., 2019).

Suicidal behavior and bullying are common among young people worldwide (Tang et al., 2020). Bullying is associated with both suicidal ideation and suicide attempts during adolescence (Holt et al., 2015; Koyanagi et al., 2019; Tang et al., 2020). A small increase in the risk of death by suicide in adulthood is observed among those who are frequently bullied during childhood (Geoffroy et al., 2023). Suicide is the third leading cause of death among 15-to 29-year-olds (World Health Organization, 2024a).

Exposure to bullying and cyberbullying is strongly associated with loneliness (Madsen et al., 2024). As seen in a study using PISA data from 51 countries, bullied students experience a lack of school belonging, poor academic performance, social integration problems, and feelings of loneliness (Yu & Zhao, 2021). As observed in a study among Nordic adolescents, the combination of cyberbullying and traditional bullying appears to exacerbate a decline in life satisfaction (Arnarsson et al., 2020).

*Definition of bullying*

Bullying involves an imbalance of power, where a person or group subjects a person or people to negative actions to intentionally cause harm or discomfort through repeated aggressive behavior over a long period of time (Olweus, 1994). Bullying can be verbal or physical and can take the form of name calling, silently “making faces”, or physical contact. It can also be direct or indirect (Olweus, 1994).

More recently, bullying has been defined as aggressive targeted behavior that harms an individual during an existing power imbalance, with emphasis placed on the frequency, intensity, and perceived harm to the victim (Volk et al., 2014). Young people’s own definition of bullying includes a criterion of health consequences alongside repetition and power imbalance. Consequently, a single but hurtful or harmful incident can be perceived as bullying, even if traditional criteria are not met (Hellström et al., 2015). Somewhat recently, the United Nations Educational, Scientific and Cultural Organization (UNESCO, 2024, 3) has presented a new definition of school bullying as *unwanted aggressive behavior that is repeated over time and includes an imbalance of power or strength that causes physical, social, and emotional harm to the targeted individuals or groups, and the wider school community harm to the targeted individuals or groups, and the wider school community.* Showing an alarming increase from 2018 to 2022 (World Health Organization, 2024b), cyberbullying refers to the use of information and communication technologies to intentionally and repeatedly harm, harm, or embarrass a specific target (Peter & Petermann, 2018).

*Causes of bullying*

Boys have a greater tendency to engage in more direct, physical forms of bullying (Cook et al., 2010; Crick et al., 1997) and be more likely to bully than girls. The likelihood of being a bully increases from grade to grade until around age 14, when such likelihood starts to decrease (Álvarez-García et al., 2015). Girls have a greater tendency to engage in relational aggression that is intended to harm someone’s relationships or social status (McGrath, 2006; Underwood, 2003) or more indirect or subtle forms of bullying, e.g., eye rolling, the spreading of secrets, whispering, and non-verbal exclusionary signals (Underwood, 2003). Relational aggression may often be overlooked because it is not considered “typical” bullying (Underwood, 2003).

New forms of bullying have emerged with the rise of new technology, including sound-based tactics and non-verbal social exclusion (Kowalski & Limber, 2013). Girls often engage in social cyberbullying and sound-based harassment, e.g., exclusion from online groups or the sending of hurtful messages or posting of content designed to harm social standing, including audio snippets (Kowalski & Limber, 2013). Cyberbullying is more common among girls than among boys (Klomek et al., 2007). Girls moreover often use sound-based teasing, which can include imitating someone’s voice, mocking laughter, or subtle inside jokes involving sound cues (used to ridicule or isolate peers) (Kowalski & Limber, 2013).

Obesity increases the risk of being a victim of bullying or being a bully (Álvarez-García et al., 2015). Low or high levels of social competence and self-esteem can predict an increased risk of bullying. Being friends with bullies, low school performance, and disinterest in studying are also risk factors for becoming bullies (Álvarez-García et al., 2015). Overweight and obese adolescents are significantly more likely to be bullied than those with a normal weight (van Geel et al., 2014).

As observed in a study on twins, there may be a genetic contribution to perpetrating bullying and being bullied (Johansson et al., 2022). Additionally, those who defend victims of bullying are more likely to be bullied by the same perpetrator (Huitsing et al., 2014). Young people can experience bullying as a means to show power, strength and superiority and become “visible” and validate oneself in a group (Caravita et al., 2020). Various behavioral and physical characteristics may underlie young people becoming victims of bullying (Caravita et al., 2020). Certain temperament traits in early adolescence could reflect difficulties in self-regulation and be linked to bullying others in middle adolescence (Farrell & Vaillancourt, 2019).

*Protective aspects and prevention work against bullying*

Informal peer involvement (e.g., group discussions) and the dissemination of information about bullying to parents/guardians (e.g., leaflets) as part of anti-bullying programs might be significantly associated with increased effectiveness in reducing exposure to and perpetration of school bullying (Gaffney et al., 2021b). Friendships with older students can also protect younger children from school bullying; thus, peer support can improve children’s school experiences (Zhu, 2023).

Support from teachers and peers may play a crucial role in resilience against bullying among adolescents (Yang et al., 2023). Adolescents who experience peer victimization can benefit from perceptions of emotional support from best friends, and intimate friendships between victimized girls may promote increased social anxiety, depressive symptoms, and maladaptive coping (Schacter & Juvonen, 2020).

Among adolescents exposed to victimization, having more friends may be associated with increased friend support (Turanovic et al., 2023). Victims who have at least one defender at the beginning of a school year feel more connected at the end of the school year than those who do not (Laninga-Wijnen et al., 2023). Victims who are defended may even experience more positive emotions and less depression, social anxiety, and loneliness than those who are not (Ma & Chen, 2019).

An ideal classroom might comprise a teacher who is perceived to be highly effective in combating bullying and does not need to exert much effort to resolve bullying situations (Veenstra et al., 2014). Important family factors regarding the risk of perpetrating bullying during adolescence include parental interest in children’s schoolwork, activities, friendships, parental limits on behavior, parental trust, closeness and communication, perceived parental emotional support, or transmitted parental values and attitudes (Álvarez-García et al., 2015). High academic achievement and coping strategies for stress may reduce depressive symptoms among young adults who are victims of bullying (Hemphill et al., 2014).

School-based anti-bullying programs can be effective in reducing both bullying and victimization (Gaffney et al., 2021a). Cyberbullying prevention programs may not only be effective against cyberbullying but also against traditional bullying (Chaux et al., 2016; Garaigordobil & Martínez-Valderrey, 2015). Youth-led programs might be effective in preventing peer aggression in school settings, and could significantly increase students’ knowledge of bullying (Connolly et al., 2015).

Specifically, the existing programs may be effective in preventing bullying. For example, the KiVa school-based anti-bullying program, a program with demonstrated efficacy (Huitsing et al., 2020; Nocentini & Menesini, 2016), might, regardless of gender, initial empathy, status, or bullying level, regardless of classroom bullying norms or school type, increase students’ affective empathy (Garandeau et al., 2022). Empathy training programs and teachers being physically present during an intervention could lead to significant increases in empathy and decreases in verbal bullying (Palade & Pascal, 2023).

Although bullying prevention programs may have desirable effects, such effects can be particularly weak among adolescents and the proposed solutions may be based on unclear assumptions (Salmivalli et al., 2021). Bullying appears to be prevented effectively up to seventh grade, but the effect for older teenagers might be reduced if not absent (Yeager et al., 2015). In addition, the KiVa program may be less effective in reducing bullying among bullies with high social power within the peer group than among less popular bullies (Garandeau et al., 2014).

Both individual and environmental factors should be considered when seeking to combat bullying effectively through anti-bullying interventions (Johansson et al., 2022). It is even more important that young people’s views on the causes of bullying and how bullying can be prevented be investigated so that anti-bullying interventions can be further developed.

**Aim of the study**

This study aimed to explore the causes of bullying among young people, and the protective mechanisms and preventive work that can promote well-being, as seen from young people’s perspectives. Two key objectives steered the research, expressed here in the form of research questions:

1. What are young people’s perspectives on the causes of bullying?
2. What young people’s perspectives on the protective mechanisms and preventive work that can promote well-being among young people?

**Materials and methods**

This study used a qualitative and exploratory design. The Consolidated Criteria for Reporting Qualitative Research (COREQ) checklist was used as a guideline for reporting the study (Tong et al., 2007).

*Sampling, setting and recruitment*

The study participants consisted of 35 young people in Finland (ten men, 25 women) aged 17-28. Participants were reached through both strategic and self-selection. Information about the study was distributed, for example, through social media, and young people were given the opportunity to contact researchers to participate. Some participants also experienced the snowball effect.

*Data collection and analysis*

Semi-structured interviews were used as data collection methods. Qualitative content analysis, according to Graneheim and Lundman (2004), was used for data analysis. To obtain an overall view of the data, the interviews were first read repeatedly, and then the text was analyzed thoroughly to find meaning units, which were subsequently condensed to create codes and subcategories. The meaning of the data was conveyed in the form of main categories. For an example of this analysis, refer to Table 1.

*(Table 1 near here)*

*Ethical considerations*

The guidelines of the Finnish National Board on Research Integrity TENK (TENK, 2023) were followed. In accordance with ethical research guidelines in Finland (TENK, 2023), participants above 15 years of age do not require guardian consent or assent. Written informed consent was obtained from and verbal information about the study given to all participants before the interviews were conducted. Information about the study’s purpose, confidentiality, voluntary participation in the study (i.e., the right to discontinue participation at any time), and the intention to publish the results were communicated to the participants both orally and in writing. An ethical license was granted [13/2024] by the Research Ethics Committee at Åbo Akademi University in Finland.

During the interviews, the researchers conducting the interviews sought to acknowledge any eventual participant distress. Participants were allowed to share their views and experiences as they desired; they were reminded of their right to decline to answer questions and were allowed breaks and/or periods of silence as needed. Some participants expressed gratitude for being given the chance to express their thoughts and feelings about the topic.

**Results**

Three main categories were generated: (1) Causes of bullying, intervention, and mitigation aspects; (2) Protection against the harmful effects of bullying and loneliness; and (3) Prevention and promotion of well-being. Nine subcategories were identified in this study. For an overview of the results, see Table 2.

*(Table 2 near here)*

Causes of bullying and intervention and mitigating aspects

This main category pertains to young people’s perspectives on the causes of bullying, interventions, and mitigation aspects. Three subcategories emerged: *To deviate from the norm or personal difficulties*, *Intervention by schools and adults,* and *Obstacles to intervention and action.*

*To deviate from the norm or personal difficulties*

According to the study participants, the most prominent reasons for bullying were being considered different or somewhat different from most other young people in a school setting or related to the appearance or style of dress. For example, participants mentioned being bullied because of their body size and/or shape, acne, teeth, or overweight. *But I remember myself from secondary school that you should of course be exactly like everyone else. You of course like didn’t get to... stand out from the crowd. If you were a bit different then you could probably be ostracized or bullied because [you] were a bit different* (P26).

Other causes of bullying could include the bully’s own personal difficulties or insecurities, or that the bully had “something against” *I think that bullying can of course also come from some kind of insecurity from the bullying child, the child perhaps doesn’t have it so good at home. Perhaps adults should be able to deal with their problems and in that way convey a better image to the child* (P34). Another reason was the bullies’ jealousy of the bullying object. *...several of them came and ...explained the reason why they were bullying – that they were a little jealous and so on...* (P19).

Other reasons, for example, were that the bullied had said or done something for which they were bullied, the bullied was lonely or quiet at school, was good at something, or had failed at something, or the bullied had been involved in an intervention against bullying. Moreover, other causes could include the bullied person’s personality or linguistic affiliation being atypical or different.

*I remember that the Finnish speakers kept together better and didn’t let us Swedish speakers into the [group] and we felt ostracized and left out, they didn’t talk to us or involve us in anything. Sometimes they also made fun of us for being Swedish-speaking precisely because we didn’t understand everything when it was in Finnish and so on. We got to hear words of abuse...* (P9).

Participants even mentioned family background as a possible cause.

*I’ve never really figured out exactly what they thought was wrong with me but it was probably that they thought I perhaps didn’t have the right clothes, I didn’t behave exactly as they behaved and that I’m from a family that belongs to [a specific religious group]. I think it also meant that I became a bit ostracized because just that particular [religious group] isn’t very popular in the secondary school I went to* (P25).

*Intervention by schools and adults*

Most participants who experienced bullying perceived that any eventual intervention or help received was insufficient or unhelpful. Some participants mentioned a specific school-based anti-bullying program (KiVa), on which their opinions were divided. Some participants revealed that their positive experiences of teachers and/or school interventions were less frequent than their experiences of “unaware” or uninformed teachers or help. *In primary school I went to the counsellor, but that probably didn’t help at all. It was a little like they blamed you* (P14).

Some participants mentioned that they had received professional help such as therapy, counselling, and meetings with psychologists or counsellors. Some participants explained that their young age hindered seeking timely help; they did not seek help. …*I was silent about so many things because I was afraid* (P29). Professional help and colleagues were supported by other participants. *When you were in it, then there was no help. ...no, actually not. But afterwards then therapy has been the biggest help, of course so have workmates given support too..., but ...it hasn’t helped so much* (P22). Some participants considered their experience of professional help useful, whereas others perceived it as not useful.

*Obstacles to intervention and action*

More than half of the participants found it difficult to talk about bullying during their childhood and adolescence. *When I was younger then I didn’t tell my parents everything, because... firstly then perhaps I didn’t always realize that it was wrong... and secondly perhaps I just felt that I couldn’t cope... like I felt like I didn’t want to then* (P1).

About half also said that they found it difficult to intervene and report bullying, with the most mentioned underlying reason being that they feared becoming victims. Some also mentioned the power imbalance inherent in bullying as a reason they did not dare intervene or act when they witnessed bullying. *Of course the rest of us could intervene and speak out, but I think most and even I were too cowardly to do it so we didn’t dare, afraid that we then would also be bullied* (P9). Approximately half of the participants mentioned that they had provided support, intervened, or took action at some point when they had witnessed bullying. *I have always had such a view on it that I have zero tolerance for bullying and ...if I see or hear that someone is treated unfairly. Whether it’s about racism or about appearance, personality, various attacks or so, or physical I have always intervened* (P7).

Protection against the harmful effects of bullying and loneliness

This main category relates to the factors that young people regard as protecting themselves from the possible harmful effects of bullying and loneliness. Two subcategories emerged: *Close and secure relationships as support* and *Change of environment, school transitions and own characteristics and actions.*

*Close and secure relationships as support*

In all interviews, participants described some type of close, trusting, or secure relationship as being protective against the harmful effects of bullying and loneliness. Close and good family, parental, and friendship relationships were most commonly described. One strategy to alleviate bullying is to spend a lot of time with family members. *I spent a lot of time with my family... I’m really close to them. And it perhaps helped, I had them... I could always rely on them* (P35). Participants also emphasized that bullying and loneliness were closely linked, but that relationships could provide relief.

*I think that ... [bullying and experiences of loneliness] have a big connection. For me ... I’ve been lucky to make so many friends, then I haven’t even though I’ve been a little bullied then I’ve still had a lot of friends outside ... Even if I was like bullied then I wouldn’t say that I felt lonely. I was still lucky to have a lot of friends that I am really grateful for* (P8).

Even mentioned were relationships with other bullied people, colleagues, or close teachers; socializing over the Internet; and contact with people via social media. *I have always had the computer at home and through it been able to socialize with friends, so I have not felt any feelings of loneliness because of this [bullying]* (P24). Participants described how anger could arise as a result of bullying. *The anger was strong and it took a long time before I let go of it, maybe at the age of 17-18, actually it was probably when I found [my] girlfriend. ... Yeah, I got to be myself and she liked me as I was. I didn’t have to pretend to be anything else* (P4).

*Change of environment, school transitions and own characteristics and actions*

In some interviews, environmental changes emerged as a protective factor against bullying and loneliness. Some participants said that leisure activities could provide a safe haven from bullying provided that bullies were not present. *I played a lot of soccer, so there you never had to worry that there would be bullying, there you were able to focus on the soccer* (P24). Some participants experienced changing schools or transitioning between school stages as positive events. *Actually [bullying] was of course at its worst in ninth grade and then there of course wasn’t so much left of school anymore and then it was summer and then you started at a new school and got a fresh start so I think that made the situation better* (P23).

Personal qualities such as positivity, self-respect, being good at sports, being brave, and one’s own beliefs were even mentioned as helpful with experiences of bullying and could provide resilience, according to the participants.

*...(I) was one of the key players which perhaps led to they didn’t dare in the same way bully me and I never let them stop me...I just like kind of anyway ran my own race...I didn’t let it get me down because for me it was a little like that at practice then if someone had been a little mean then quite honestly I can say that perhaps I went a little harder [during defensive play]...* (P5).

Actions to end bullying also emerged. Some participants who had been bullied reported that they took matters into their own hands and confronted their bully.

*Yeah... it probably became better when I confronted the person in question... I told [the person] that I had seen the [bullying] messages... [The person] became very sad and asked for forgiveness... but it left me with severe scars for the rest of secondary school*

*... I had a hard time trusting that the people in the group [actually] wanted to spend time with me...* (P16).

Another participant used physical self-defense to counter and stop their bullying. Although such actions did not always stop bullying, some participants perceived that it alleviated the situation. One participant who had reacted to a bullying incidence with a physical counteraction said that nothing else would have helped at the time, while acknowledging that physical action is not a solution. *The only times that bullying problems have been solved is when I’ve fought those who have bullied me... Like I’m still not talking about any [serious fighting]...I instead mean only a little wrestling like young boys do...* (P4).

Prevention and promotion of well-being as solutions

This main category encompasses young people’s perspectives on solutions to bullying, and the prevention and promotion of well-being could look like. All participants emphasized the importance of prevention, and the role of adults was highlighted as central to the prevention of bullying. The promotion of well-being as a way to prevent bullying also permeated the participants’ statements. Four sub-categories emerged: *Safe meeting spaces for inclusion and collaboration as well as professional support; Community, security and openness; Upbringing and dissemination of knowledge;* and *Engagement and intervention*.

*Safe meeting spaces for inclusion and collaboration as well as professional support*

Resources, funding, and information are often emphasized in the role of society in bullying prevention. However, improved societal resources and allocation were not considered sufficient, and the importance of collaboration between various actors emerged in most participants’ statements. Almost all participants emphasized resources related to society’s role in addressing bullying and loneliness. For example, participants mentioned that society should provide schools with tools (e.g., anti-bullying curricula), ensure well-being, not reduce educational resources, provide training on bullying, and ensure that all young people can access free leisure activities. *But society can of course ensure then, for example the school ... the environment and all the facilities... all other activities... work* (P30).

Many participants highlighted the importance of professional help in cases of bullying, such as counselling, school health services, social workers, psychologists, and school coaches. *I think that the counsellor and psychologist have a really nice role in student care, and often children can feel that it can be easier to talk to a counsellor than to a teacher* (P12).

Participants viewed hobbies or gathering places for young people as important settings that could function as places of safety and counterbalance any perceived loneliness at school. Hobbies or gathering places are considered to counteract loneliness by giving young people the opportunity to participate in health promoting activities.

*Society if you think like that on the municipal level, then we need to open more places for young people. ... where you can safely socialize with other young people... We need... hobbies that suit everyone. There needs to be opportunities to socialize with different people, which means that if you feel lonely at school, which sometimes perhaps is unavoidable, no matter how we work with acceptance, then you know that ‘hey I have my hobby twice a week and there I have friends’. We need to enable more places for young people to meet around different activities* (P3).

Other aspects mentioned as belonging to societal responsibility include inclusion and integration into society, gender equality work, and anti-bullying networks.

*Community, security and openness*

Most participants emphasized the importance of the community in reducing bullying and loneliness. The community was described in terms of family, friends, peer support, being seen and heard, hobbies, the inclusion of lonely people, teachers’ and schools’ responsibility to strengthen the community, and society’s responsibility to support the building of the community.

*Then I think that both in school environments and in hobbies and sports more should be done to reduce exclusion. That if teachers see students who are lonely... that they talk to them and try to fix it and prevent it. I myself think it’s important to involve everyone so that no one becomes left out and ostracized...* (P9).

The importance of a sense of security permeated most participants’ views on solutions to bullying, mainly relevant to home and school settings; however, leisure time and community were also mentioned. Among other things, a sense of security at home was considered to contribute to resilience against bullying, including familial openness and trust, daring to and being able to talk to, and accepting parents.

*It is very important that parents should create an open environment in the home and raise their children in that way so that they dare to come and discuss... And if bullying is experienced... that they then dare to ...tell [their parents] about this... especially if you are a little younger if you are exposed to bullying then it is likely of course [your parents] you get the most support from* (P16).

A sense of security at school included the presence of teachers, a pleasant school environment, sufficient staff, zero tolerance for bullying, the behavior of peers, and getting to know others around you. One participant stated that peers should create a more positive and accepting attitude toward others because the community contributes to security and vice versa. *... have in some way a generally more accepting and positive attitude toward others in order to create a safe atmosphere... But it’s difficult, it’s of course hard when everyone themselves is unsure* (P10).

*Upbringing and dissemination of knowledge*

Most participants emphasized upbringing as a way to reduce bullying. The roles of the home and parents were often highlighted, but the teachers’ and coaches’ responsibilities were also mentioned. One participant stated that the parents should be present and act as role models. *Yes... be present ... talk about what is right and wrong and be an example...* (P17).

Most participants mentioned the importance of disseminating knowledge and information to schools, workplaces, leisure activities, and society. Participants referred to school-based action programs against bullying (e.g., KiVa), the prevention of bullying and loneliness at the community level, and the importance of spreading knowledge about bullying. To this end, schools should be active according to participants. *First of all then, talk about bullying and the consequences of bullying. And like actually some talk about how self-image is changed by bullying and that that is very negative* (P13). Some participants also highlighted the use of experts based on their experience. One participant stated that society should support efforts to prevent bullying and to contribute to the spread of knowledge. *Raise awareness of bullying ...it could probably be talked about even more in society. And just listen to those who have been bullied and how it has affected them later in life* (P35).

*Engagement and intervention*

While all participants emphasized the importance of preventive work to prevent bullying, most also mentioned intervention as an important factor in reducing bullying and loneliness. Interventions included intervening, standing up for the person being bullied, reacting to, or telling someone about bullying. Regarding who should intervene, adults were mentioned first, but participants also emphasized that peers or friends played an important role.

*I think peers and friends need to be attentive... and yes actually take that step of speaking out if something is wrong... and make contact with someone who like actually can influence [the situation]. And just that... If you feel that you don’t dare confront the bully you can contact teachers, principals or managers...* (P28).

The school staff, teachers, and parents were mentioned by the participants as the adults who should intervene. Adults (in principle, adults) were referred to as coaches and staff. Participants primarily emphasized the role of adults and that adults bore the greatest responsibility for intervening and reducing bullying because they held different positions of power.

*It’s* *[important] to discuss and there can also... other than friends, there is also perhaps just this with the school [it] can intervene and the parents can intervene, but the friends don’t have... so much power over a bully, because... yes they are of course on the same level, they are the same age, they have the same view on the matter even though they perhaps don’t bully...* (P30).

Half of the participants also highlighted the importance of parental commitment to their children as an important factor in reducing bullying. Parents’ engagement was explained by including issues related to schooling and leisure, listening, attendance, support, perception, and participation.

*Parents should talk to their children, ask questions and be available. Ask about what is difficult in order to be a support and show that the problem is taken seriously. ...tell that they know what is going on and that they will do everything they can to stop the bullying. Then the parents surely should contact the teacher ... and say ...it must stop immediately* (P27).

Only a few participants reported the consequences of bullying. Among other things, changing schools, stricter consequences, punishments, and changes in criminal liability were mentioned in relation to the measures that the bullies should face. Participants noted that it is probably not always beneficial for a person who has already been bullied – an excluded and lonely young person – to be transferred to a new school because this could increase their problems.

*I also think that it’s primarily those who bully who should change schools at least that main bully, because often it’s of course the case that there is one or a few main bullies and the rest just kind of follow along, so I think that if that main bully would switch schools or in some other way be removed from the equation then it would at least considerably reduce that bullying* (P21).

**Discussion**

We explored the causes of bullying among young people, and the protective mechanisms and preventive work that can promote well-being from young people’s perspectives. From the results, we conclude that participants perceived many different reasons for bullying. Most prominently, the bullied person was considered different or different in some way (cf. Hopkins et al., 2013; Thornberg, 2018; Wójcik, 2018) and bullying was linked to appearance (e.g., acne, being overweight, body size/shape, teeth) or clothing style (cf. Prince et al., 2024). Previous studies have also shown that overweight or obese children and adolescents are at greater risk of being bullied (Ashrafi et al., 2020; Çalışkan et al., 2019; Cheng et al., 2022; Hammar et al., 2020; Merrill & Hanson, 2016). Self-perceived overweight and frustration with appearance have also been related to young people’s experience of being bullied among young people (Lin et al., 2018).

The bullies had something against them, and personal difficulties or insecurities were also highlighted by the participants in this study as causes of bullying. Other research has shown that in long-term bullying cases, there is a complex pattern of interaction during which stigmatizing processes are created, through which bullying is considered natural and justified through the blaming and dehumanization of the victim (Thornberg, 2015b). Moreover, it has been found that young people are strongly inclined to believe that bullies want power or status and have psychosocial problems and that victims of bullying are different, odd or deviant in some way (Thornberg, 2015a). Students have even been found to believe that bullies have their own problems (Mischel & Kitsantas, 2020). Bullies’ jealousy has also been highlighted as a major cause of bullying, which was mentioned by the participants in this study (cf. Geng et al., 2022; Reason et al., 2016; Villanueva-Moya et al., 2023; Wang et al., 2019). For example, participants even mentioned that the bullied person had said or done something for which they were bullied or was alone or quiet at school (cf. Ybarra et al., 2019), that the bullied person was good at or had conversely failed at something, and that the bullied person’s personality was atypical or somehow different (cf. Strindberg et al., 2020). Additionally, participants in this study stated that intervening in bullying could lead to being bullied, but others have seen that defending victims of bullying is not significantly associated with future victimization (Malamut et al., 2023).

Family background and different linguistic affiliations were also noted as the causes of bullying. This is supported by previous research, in which the tendency to attribute certain specific characteristics to a certain origin or ethnicity is at the heart of bullying has been seen (Mazzone et al., 2018). However, earlier research is somewhat unclear on this matter; some have found that any reason can lead to bullying (Forsberg & Thornberg, 2016).

We found that interventions by schools and adults were considered insufficient or unhelpful by most participants who had experienced bullying. Participants also referred to a specific school-based anti-bullying program (KiVa); however, their opinions were divided (Mischel & Kitsantas, 2020). In earlier research, one study found only short-term positive results associated with KiVa regarding student well-being and the reduction of bullying (van der Ploeg et al., 2016), while in another study, most students stated that they had never participated in programs that specifically dealt with bullying prevention (Ybarra et al., 2019). Some participants in this study also mentioned the experiences of unaware or uniform teachers (cf. Evans et al., 2017). The importance of school support and resources has been highlighted in previous research, and teacher and/or classmate support has been seen as a key protective factor associated with a lower likelihood of becoming a victim of bullying (Jaskulska et al., 2022).

Some participants in this study received professional help to alleviate their bullying experience. However, previous studies have been mixed in this regard. Some studies have shown that exposure to bullying is associated with psychiatric care (Evans-Lacko et al., 2017; Garaigordobil & Machimbarrena, 2019) while others have found that a significant proportion of bullied young people do not use any psychiatric care services (Islam et al., 2022). In this study, some participants found professional help useful, while others did not, and some participants mentioned that colleagues had provided support (cf. Karatuna, 2015).

Over half of the participants found it difficult to talk about bullying during childhood and adolescence (cf. Boulton et al., 2017; Danielson & Jones, 2019; Yablon, 2020). Previous research has also found that only half of those who have been bullied had told someone, and significantly fewer had told an adult, and that it is more common to tell a home-based adult about bullying than a school-based adult (Blomqvist et al., 2020). Others have found that difficulties in socializing and receiving social support are critical factors linked to reduced well-being and psychological distress among young people (Filia et al., 2025). In this study, about half of the participants found it difficult to intervene and report bullying, and most participants mentioned a fear of themselves becoming victims of bullying, while some participants noted a power imbalance (cf. Forsberg et al., 2018; Strindberg et al., 2020; Thornberg et al., 2012, Thornberg, 2015b). Approximately half of the participants said that they had provided support, intervened, or acted at some point when they had witnessed bullying. Earlier research has shown that most adolescents report participating in defensive behaviors (Evans et al., 2017; Lambe et al., 2017).

All participants mentioned that some type of close, trusting, or secure relationship, with family, parental, or friendship relationships most commonly described, could protect against the harmful effects of bullying and loneliness (cf. Hellfeldt et al., 2020; Holfeld & Baitz, 2020; Yang et al., 2023). Spending a lot of time with the family is one strategy used to alleviate bullying. Previous research has shown that higher levels of family belonging and support can reduce exposure to bullying (Fan & Meng, 2022) as well as peer support (Biswas et al., 2020). In this study, participants highlighted that bullying and loneliness were strongly linked but that relationships could provide relief. Earlier research has also demonstrated a link between bullying and loneliness (cf. Eid et al., 2023; Madsen et al., 2024; Strindberg, 2023).

We found that changes in the environment could protect young people from bullying and loneliness. Some participants mentioned that leisure activities could constitute a safe haven from bullying, albeit only if bullies are not present (cf. Bjereld et al., 2024; Lidberg et al., 2024). Changing schools and transitioning between school stages were also considered positive events by some participants (cf. Frisén et al., 2012; Låftman et al., 2024). Personal qualities (positivity, self-respect, good at sports, and brave) and beliefs were even noted as having helped against bullying (cf. Munawaroh et al., 2024). Some participants even described taking their own actions to resolve bullying, for example, confronting the bully or physical counteraction (cf. Bouchard et al., 2018; Evans et al., 2017; Lidberg et al., 2024; Ybarra et al., 2019), while others noted that such actions alleviated bullying even if it did not stop.

Relevant to society’s role in the prevention of bullying, nearly all participants emphasized the need for resources, funding, information, and collaboration among various actors. Previous research has found that successful school-based prevention of interpersonal violence is based on strong, trusting, and collaborative relationships (Edwards et al., 2023), and that anti-bullying interventions involving different parties build more effective awareness (Hikmat et al., 2024). Participants in this study stated that schools should be given anti-bullying tools, well-being and educational resources should be ensured, bullying training should be offered, and access to free leisure activities should be ensured. Previous research has indicated that many schools need support when starting and initially implementing anti-bullying programs (Sainio et al., 2020). Others have found, for example, that social engagement and belonging are important elements in prevention programs against cyberbullying, arguing that school staff should be trained to recognize symptoms in youth that could indicate a lack of friends or social activities (Stegmair & Prybutok, 2024). Previous research has shown that bullying is related to sedentary leisure time (Zapata-López et al., 2024), supporting the importance of arranging free leisure activities for both children and young people.

Many participants highlighted the importance of professional help in bullying cases. Previous research has shown that when seeking or using mental healthcare, bullied young people are more likely to use online and health services and less likely to use school and telephone services (Islam et al., 2022). Others have seen that only a minority of young people would use professional support if they had any concerns (Noret et al., 2020) or that bullying decreases most consistently in schools where staff report increased referrals to counselling and other support for victims of bullying (Ramirez et al., 2024). In this study, participants stated that hobbies or gathering places for young people are important and could act as places of safety or counterbalance to bullying. This is supported by previous research in which physically active adolescents were shown to be less likely to suffer from social isolation (dos Santos et al., 2021). Previous research has also found a positive correlation between physical activity during leisure time and mental health (White et al., 2017). Participants in this study even noted inclusion and integration into society, gender equality, and anti-bullying networks as part of society’s responsibilities. Others have found that promoting inclusion is essential in anti-bullying work (Laitinen et al., 2020; Virrankari et al., 2020) and emphasize the importance of taking into account all environments in which children and young people spend their free time and ensuring that there are equal opportunities to be part of society, which can take the form of free and accessible leisure activities (Virrankari et al., 2020).

Most participants in this study underscored the importance of the community in reducing bullying and loneliness and mentioned several ways to strengthen the community. Previous research has also highlighted that it is the failure of parents, schools, and society to satisfy basic human needs, including a sense of belonging that underlies bullying, with a lack of attachment to parents, unavailable parents, or lack of attention in the home being examples (Wilkinson, 2017). Others have observed that school staff consider the establishment of strong student-to-student relationships and team spirit among students important in combating cyberbullying (Masoumi et al., 2024).

From the results, we found that the importance of a sense of security was included in most participants’ perceptions of solutions to bullying, mainly connected to home and school settings, leisure time, and community. Safety and security are repeatedly mentioned in the Finnish National Agency for Education’s approach to the prevention of bullying in education and training and are relevant to areas such as systematic management, planning of interventions and environments, cooperation during interventions, and staff competence (Opetushallitus, 2025). Others have also found that a sense of security is a protective factor against life satisfaction among young people who have been bullied (Varela et al., 2022).

Relevant to a sense of security in the home, participants in this study mentioned, among other things, familial openness and trust, daring to and being able to talk, and parents who were accepting of contributing to resilience against bullying. Previous research has shown that greater security in relation to parents and peers is associated with less involvement in bullying and greater defense of a victim (Murphy et al., 2017). Others have found that young people turn to acquaintances rather than professionals when they need support and that certain conditions (e.g., trust and familiarity) facilitate storytelling, while emotional support (e.g., empathy, presence, and that others care and see you) is most valued by young people (Camara et al., 2017). Most adolescents deal with bullying by talking to their parents (Evans et al., 2017), whereas more boys than girls state that they have no one to talk about their concerns (Noret et al., 2020).

Regarding the sense of security at school, the participants in this study mentioned, for example, teachers’ presence, a pleasant school environment, sufficient staff, and zero tolerance for bullying (cf. Fisher et al., 2018). As seen in previous research young people chose which adult they disclose bullying to, with a “suitable” adult being one who is deemed able to help, responsible and consistent and who is considered to take the matter seriously (Wójcik & Rzeńca, 2021). Supportive conversations with teachers and effective interventions are important (Wójcik & Rzeńca, 2021). Participants in this study also mentioned the behavior of peers and getting to know others around them in a school setting. Others have also observed the importance of class cohesion as a protective factor against cyberbullying (Parisse et al., 2024).

The importance of prevention and the role of adults were further emphasized in this study as key to the prevention of bullying (cf. Celik et al., 2023). Most participants highlighted upbringing as a way of reducing bullying. Participants often stressed the role of home and parents and mentioned the responsibility of teachers and coaches. Previous research has found that children learn from adults, and that many bullies have negative childhood experiences, for example, due to a lack of relationships with parents or bad role models (Wilkinson, 2017). Others have found that children and adolescents neglected by their parents are more likely to engage in bullying and cyberbullying (Gan et al., 2023; Wang et al., 2017; Wang & Jiang, 2022; Wilkinson, 2017) or be subjected to bullying (Cuesta et al., 2021). Bullies have also been found to have worse subjective well-being than non-involved individuals (Arslan et al., 2021). In this study, most participants mentioned the importance of disseminating knowledge and information, such as school-based action programs against bullying and community-level prevention of bullying and loneliness. Some emphasized the use of experts based on their experience. Previous research has shown that school staff highlights the importance of funds for prevention resources and the desire for uniform and transparent guidance for bullying prevention and intervention, and that every school, by law, should have a bullying prevention program and the creation of a culture of respectful actions by teachers, parents, and students (Lechner et al., 2023). The participants in this study even mentioned the importance of spreading knowledge about bullying, and that schools should be active (cf. Celik et al., 2023).

Engagement and intervention were also important factors. All participants noted the importance of preventive work in preventing bullying and most also mentioned intervention as an important factor. Regarding who should intervene, participants first mentioned adults but even emphasized that peers or friends played an important role. However, participants principally highlighted the role of adults, noting that they bear the greatest responsibility for intervening and reducing bullying because, in comparison to young people, they hold a different position of power (cf. Wood et al., 2017). Previous research has also shown that young people believe that peers and even themselves should intervene when they witness bullying, although they also report that peers are most often bystanders in school bullying cases and do not intervene (Thornberg et al., 2018).

Half of the participants underlined the importance of parental commitment to or engagement with their children (related to schooling and leisure, listening, attendance, support, being observant, and participation) in reducing bullying (cf. Cassidy et al., 2018; Mehari et al., 2018). Previous research has shown that parental phubbing is associated with a greater risk of adolescents committing cyberbullying acts at an early age (10–12 years) and experiencing cyberbullying victimization at a later age (≥ 15 years) (Elboj-Saso et al., 2024). In this study, only some participants mentioned the consequences for bullies, and participants stated that moving a bullied, excluded, and lonely young person to a new school is perhaps not always beneficial, because it can increase their problems. Previous studies have also shown that for young people, punishment is not the most suitable solution for bullying (cf. Cassidy et al., 2018; Wood et al., 2017).

*Strengths and limitations*

The sample in this study was predominantly female, which may have affected the generalizability of the findings. Had the sample included more male participants, the results might have been differed slightly. Information about the study on social media may have contributed to the participants’ interest in talking about the study subject and thereby the self-selection method, which might have contributed to a rich dataset. However, the participants were from varying ages, backgrounds, life circumstances, and experiences, which contributed to the study’s higher credibility and transferability. With the help of a semi-structured interview guide and detailed participant answers and descriptions, broad questions yielded rich data. Reliability was verified by ensuring agreement between all study researchers and including illustrative and expressive quotations from the dataset. A consensus on the results was attained through discussions between the researchers.

**Conclusion**

We observed that most young people viewed themselves differently as a cause of bullying. Participants emphasized prevention and cooperation in counteracting bullying. The role of adults was highlighted, and community, sense of security, and openness were found to be important in reducing bullying. Parents’ commitment to their children and engagement in their upbringing were mentioned as being important in alleviating experiences of bullying. Some types of close, trusting, or secure relationships protect against the harmful effects of bullying and loneliness. A young person’s characteristics, abilities, and resilience, as well as changes in the environment, can even be seen as protective.

Interventions in bullying are recognized as an essential factor in reducing bullying. School-based help or interventions for bullying are often considered insufficient or unhelpful. Intervening and talking about bullying were also considered difficult. Sufficient resources and societal awareness are important. Hobbies, gathering places, and professional help are considered essential in bullying cases. The fact that bullies face consequences is often not mentioned.

The importance of close, trusting, or secure relationships should be addressed in student healthcare. Including psychiatric nurses with experience with children and adolescents could also be a more generally used school resource. Intervening and talking about bullying and fostering young people regarding, for example, acceptance, inclusion, diversity, and respect for others should be practiced in home and school settings, for which a confidential relationship with the adults involved is fundamental. Becoming accustomed as a young person to speaking about one’s mental health (feelings, thoughts, and experiences) might enable talking about difficult situations or issues (e.g., loneliness and bullying) as needed, which could even provide the strength to seek help for oneself or others or help reduce any stigma associated with mental health issues, bullying, or loneliness.

Further research could explore interventions to prevent bullying in school settings, focusing on how bullying prevention programs are implemented and how school staff and guardians perceive and work with bullying issues.

**Acknowledgements**

The authors extend their sincere appreciation to the study participants for their contribution.

**Disclosure statement**

The authors report there are no competing interests to declare.

**Funding details**

None.

**Data availability statement**

No data was shared due to ethical issues.

**Biographical note**

**Miia Hästbacka**, Åbo Akademi University, Faculty of Education and Welfare Studies, Department of Health Sciences, Vaasa, Finland. PhD candidate in Health Sciences, Department of Health Sciences, Faculty of Education and Welfare Studies, Åbo Akademi University, Vaasa, Finland. Research has focused on adolescents’ and young adults’ mental health, bullying, and loneliness. E-mail: [miia.hastbacka@abo.fi](mailto:miia.hastbacka@abo.fi) ORCID: <https://orcid.org/0009-0004-2270-7338>

**Søren Harnow Klausen** is a professor of philosophy at the University of Denmark and associated with Åbo Akademi University (as guest professor 2022-2024 and from 2025 as a special researcher). He is currently the head of the research project *Wellbeing and Virtual Worlds* funded by the Independent Research Fund, Denmark. His broad research interests include the philosophy of education, well-being, *Bildung* and other notions of personal development, epistemology, phenomenology, philosophy of health and aging, philosophy and psychology of climate change, cross-cultural philosophy, and the philosophy of technology. He edited the anthology and *Developing a didactic framework across and beyond school subjects* (Routledge, 2024). E-mail: [harnow@sdu.dk](mailto:harnow@sdu.dk) ORCID: [https://orcid.org/0000-0002-2650-1861.](https://orcid.org/0000-0002-2650-1861)

**Heléne Dahlqvist,** Ph.D. in Philosophy, Senior Lecturer, Public Health Science, Department of Health Sciences, MidSweden University, Sundsvall, Sweden. Program Director, Bachelor’s Program in Public Health. The main research areas include mental health in youth, the psychosocial school environment, and gender, with a special focus on sexual harassment in school and online. Participatory research (emerging areas). E-mail: [helene.dahlqvist@miun.se](mailto:helene.dahlqvist@miun.se) ORCID ID: <https://orcid.org/0000-0003-3209-186X>

**Yulia Korzhina**, PhD candidate in Health Sciences, Department of Health Sciences, Faculty of Education and Welfare Studies, Åbo Akademi University, Vaasa, Finland. Research has focused on loneliness and ethical issues related to caring for adolescents ‘and young adults. ’ E-mail: [yulia.korzhina@abo.fi](mailto:yulia.korzhina@abo.fi) ORCID ID: <https://orcid.org/0000-0001-9307-7637>

**Amanda J. Sundqvist**, PhD candidate in Health Sciences, Department of Health Sciences, Faculty of Education and Welfare Studies, Åbo Akademi University, Vaasa, Finland. Research has focused on adolescents and young adults and their relationship with mental health and loneliness. E-mail: [amanda.sundqvist@abo.fi](mailto:amanda.sundqvist@abo.fi) ORCID ID: [https://orcid.org/0000-0001-7204-4895.](https://orcid.org/0000-0001-7204-4895)

**Emelie Käcko**, PhD candidate in Health Sciences, Department of Health Sciences, Faculty of Education and Welfare Studies, Åbo Akademi University, Vaasa, Finland. Research has focused on social media use and loneliness among adolescents and young adults. E-mail: [emelie.kacko@abo.fi](mailto:emelie.kacko@abo.fi) ORCID ID: <https://orcid.org/0000-0001-7554-1125>

**Pia Nyman-Kurkiala**, PhD in Social Sciences, Docent in Sociology, Researcher in Youth Sciences, Faculty of Education and Welfare Studies, Åbo Akademi University, Vaasa, Finland. 2020-2024 one of the project leaders at the Center of Expertise NUOSKA - Youth Work at Schools and Educational Institutions, managed by the Finnish Ministry of Education and Culture. Leaders of several research projects on, for example, the ethnic identity of minority young people, loneliness, and well-being among adolescents, and multiprofessional collaboration in a school context concerning health-promoting student care for children and youth. E-mail: [pia.nyman-kurkiala@abo.fi](mailto:pia.nyman-kurkiala@abo.fi) ORCID ID: <https://orcid.org/0000-0002-1462-9251>

**Joakim Strindberg**, PhD in Sociology, Senior Lecturer, Department of Pedagogy, Faculty of Social Sciences, Linnaeus University, Kalmar, Sweden. Research interests include loneliness and bullying. ORCID ID: <https://orcid.org/0000-0001-6098-8389>

**Jessica Hemberg**, PhD in Health Sciences, PHN, RN, Associate Professor, Senior Lecturer, Department of Caring Sciences, Faculty of Education and Welfare Studies, Åbo Akademi University, Vaasa, Finland. Subject leaders in pedagogical studies for teachers in Health Sciences Didactics. Among other projects, the leader of a research project on loneliness and well-being among adolescents and young adults and of a research project on multiprofessional collaboration in a school context concerning health-promoting student care for children and youth. Other areas of expertise include well-being, suffering, loneliness among older adults, ethical issues and compassion in caring, and ethical leadership. E-mail: [jessica.hemberg@abo.fi](mailto:jessica.hemberg@abo.fi) ORCID ID: <https://orcid.org/0000-0002-0829-8249>

*Corresponding author:

**Miia Hästbacka**, Åbo Akademi University, Faculty of Education and Welfare Studies, Department of Health Sciences, Vaasa, Finland. PhD candidate in Health Sciences, Department of Health Sciences, Faculty of Education and Welfare Studies, Åbo Akademi University, Vaasa, Finland. Research has focused on adolescents’ and young adults’ mental health, bullying, and loneliness. E-mail: [miia.hastbacka@abo.fi](mailto:miia.hastbacka@abo.fi) ORCID: <https://orcid.org/0009-0004-2270-7338>

**Data Availability**

No data were shared owing to ethical issues and the protection of the individuals who participated in this study.

**References**

Álvarez-García, D., García, T., & Núñez, J. C. (2015). Predictors of school bullying perpetration in adolescence: A systematic review. *Aggression and Violent Behavior, 23*, 126–136. <https://doi.org/10.1016/j.avb.2015.05.007>

Arnarsson, A., Nygren, J., Nyholm, M., Torsheim, T., Augustine, L., Bjereld, Y., Markkanen, I., Schnohr, C. W., Rasmussen, M., Nielsen, L., & Bendtsen, P. (2020). Cyberbullying and traditional bullying among Nordic adolescents and their impact on life satisfaction. *Scandinavian Journal of Public Health*, *48*(5), 502–510. <https://doi.org/10.1177/1403494818817411>

Arslan, G., Allen, K.-A., & Tanhan, A. (2021). School Bullying, Mental Health, and Wellbeing in Adolescents: Mediating Impact of Positive Psychological Orientations. *Child Indicators Research, 14*(3), 1007–1026. <https://doi.org/10.1007/s12187-020-09780-2>

Ashrafi, A., Feng, C. X., Neudorf, C., & Alphonsus, K. B. (2020). Bullying victimization among preadolescents in a community-based sample in Canada: a latent class analysis. *BMC Research Notes, 13*, 138. <https://doi.org/10.1186/s13104-020-04989-4>

Berger, C., & Caravita, S. C. S. (2016). Why do early adolescents bully? Exploring the influence of prestige norms on social and psychological motives to bully. *Journal of Adolescence, 46*(1), 45–56. <https://doi.org/10.1016/j.adolescence.2015.10.020>

Biswas, T., Scott, J. G., Munir, K., Thomas, H. J., Huda, M. M., Hasan, M. M., David de Vries, T., Baxter, J., & Mamun, A. A. (2020). Global variation in the prevalence of bullying victimisation amongst adolescents: Role of peer and parental supports. *EClinicalMedicine, 20*, 100276. <https://doi.org/10.1016/j.eclinm.2020.100276>

Bjereld, Y., Thornberg, R., & Wójcik, M. (2024). “Just shut out the world”: Bullied adolescents’ structured recreational and sports activities and their relationship to wellbeing and friendship. *Journal of Leisure Research*, 1–15. <https://doi.org/10.1080/00222216.2024.2396471>

Blomqvist, K., Saarento-Zaprudin, S., & Salmivalli, C. (2020). Telling adults about one’s plight as a victim of bullying: Student- and context-related factors predicting disclosure. *Scandinavian Journal of Psychology, 61*(1), 151–159. <https://doi.org/10.1111/sjop.12521>

Bouchard, K., Forsberg, C., Smith, J. D., & Thornberg, R. (2018). Showing friendship, fighting back, and getting even: resisting bullying victimization within adolescent girls’ friendships. *Journal of Youth Studies*, *21*(9), 1141–1158. <https://doi.org/10.1080/13676261.2018.1450970>

Boulton, M. J., Boulton, L., Down, J., Sanders, J., & Craddock, H. (2017). Perceived barriers that prevent high school students seeking help from teachers for bullying and their effects on disclosure intentions. *Journal of Adolescence, 56*(1), 40–51. <https://doi.org/10.1016/j.adolescence.2016.11.009>

Çalışkan, Z., Evgin, D., Bayat, M., Caner, N., Kaplan, B., Öztürk, A., & Keklik, D. (2019). Peer Bullying in the Preadolescent Stage: Frequency and Types of Bullying and the Affecting Factors. *The Journal of Pediatric Research, 6*(3), 169–179. <https://doi.org/10.4274/jpr.galenos.2018.26576>

Camara, M., Bacigalupe, G., & Padilla, P. (2017). The role of social support in adolescents: are you *helping me* or *stressing me out*? *International Journal of Adolescence and Youth, 22*(2), 123–136. <https://doi.org/10.1080/02673843.2013.875480>

Caravita, S. C. S., Stefanelli, S., Mazzone, A., Cadei, L., Thornberg, R., & Ambrosini, B. (2020). When the bullied peer is native-born vs. immigrant: A mixed-method study with a sample of native-born and immigrant adolescents. *Scandinavian Journal of Psychology, 61*(1), 97–107. <https://doi.org/10.1111/sjop.12565>

Cassidy, W., Faucher, C., & Jackson, M. (2018). What Parents Can Do to Prevent Cyberbullying: Students’ and Educators’ Perspectives. *Social Sciences*, *7*(12), 251. <https://doi.org/10.3390/socsci7120251>

Celik, H. C., Hopkins, L., & O’Reilly, M. (2023). Exploring the perspectives of Turkish adolescents on bullying: A qualitative study. *Psychology in the Schools, 60*(8), 2729–2743. <https://doi.org/10.1002/pits.22883>

Chaux, E., Velásquez, A. M., Schultze-Krumbholz, A., & Scheithauer, H. (2016). Effects of the cyberbullying prevention program media heroes (*Medienhelden*) on traditional bullying. *Aggressive Behavior, 42*(2), 157–165. <https://doi.org/10.1002/ab.21637>

Cheng, S., Kaminga, A. C., Liu, Q., Wu, F., Wang, Z., Wang, X., & Liu, X. (2022). Association between weight status and bullying experiences among children and adolescents in schools: An updated meta-analysis. *Child Abuse & Neglect, 134*, 105833. <https://doi.org/10.1016/j.chiabu.2022.105833>

Cook, C. R., Williams, K. R., Guerra, N. G., Kim, T. E., & Sadek, S. (2010). Predictors of bullying and victimization in childhood and adolescence: A meta-analytic investigation. *School Psychology Quarterly, 25*(2), 65–83. <https://doi.org/10.1037/a0020149>

Connolly, J., Josephson, W., Schnoll, J., Simkins-Strong, E., Pepler, D., MacPherson, A., Weiser, J., Moran, M., & Jiang, D. (2015). Evaluation of a Youth-Led Program for Preventing Bullying, Sexual Harassment, and Dating Aggression in Middle Schools. *The Journal of Early Adolescence, 35*(3), 403–434. <https://doi.org/10.1177/0272431614535090>

Cuesta, I., Montesó-Curto, P., Metzler Sawin, E., Jiménez-Herrera, M., Puig-Llobet, M., Seabra, P., & Toussaint, L. (2021). Risk factors for teen suicide and bullying: An international integrative review. *International Journal of Nursing Practice,* *27*(3), e12930. <https://doi.org/10.1111/ijn.12930>

Crick, N. R., Casas, J. F., & Mosher, M. (1997). Relational and overt aggression in preschool. *Developmental Psychology, 33*(4), 579–588. <https://doi.org/10.1037/0012-1649.33.4.579>

Danielson, C. M., & Jones, S. M. (2019). “Help, I’m Getting Bullied”: Examining Sequences of Teacher Support Messages Provided to Bullied Students. *Western Journal of Communication, 83*(1), 113–132. <https://doi.org/10.1080/10570314.2018.1490451>

dos Santos, A. E., Araujo, R. H. de O., Nascimento, V. M. S. do., Couto, J. de O., & Silva, R. J. dos S. (2021). Associations between specific physical activity domains and social isolation in 102,072 Brazilian adolescents: Data from the 2015 National School–Based Health Survey. *Journal of Health Psychology, 26*(13), 2626–2635. <https://doi.org/10.1177/1359105320922298>

Edwards, K. M., Orchowski, L. M., Espelage, D. L., & Temple, J. R. (2023). What Is Not in the Methods Section: Challenges, Successes, and Lessons Learned From Conducting School-Based Interpersonal Violence Prevention Research*. Journal of Interpersonal Violence, 38*(3-4), 4507–4532. <https://doi.org/10.1177/08862605221109881>

Eid, E., Fekih-Romdhane, F., Sarray El Dine, A., Malaeb, D., Hallit, S., & Obeid, S. (2023). Does Problematic Use of Social Network Mediate the Association between Bullying Victimization and Loneliness among Lebanese Adolescents? *Children, 10*(3), 599. <https://doi.org/10.3390/children10030599>

Elboj-Saso, C., Íñiguez-Berrozpe, T., Cebollero Salinas, A., & Bautista Alcaine, P. (2024). “Listen to me!” The role of family supervision and parental phubbing in youth cyberbullying. *Family Relations, 73*(3), 1568–1587. <https://doi.org/10.1111/fare.12968>

Evans, C. B. R., Cotter, K. L., & Smokowski, P. R. (2017). Giving Victims of Bullying a Voice: A Qualitative Study of Post Bullying Reactions and Coping Strategies*. Child & Adolescent Social Work Journal, 34*(6), 543–555. <https://doi.org/10.1007/s10560-017-0492-6>

Evans-Lacko, S., Takizawa, R., Brimblecombe, N., King, D., Knapp, M., Maughan, B., & Arseneault, L. (2017). Childhood bullying victimization is associated with use of mental health services over five decades: a longitudinal nationally representative cohort study. *Psychological Medicine*, *47*(1), 127–135. <https://doi.org/10.1017/S0033291716001719>

Fan, L. L., & Meng, W. J. (2022). The relationship between parental support and exposure to being bullied: Family belonging as a mediator.*Social Behavior and Personality, 50*(12), e12022. <https://doi.org/10.2224/sbp.12022>

Farrell, A. H., & Vaillancourt, T. (2019). Temperament, Bullying, and Dating Aggression: Longitudinal Associations for Adolescents in a Romantic Relationship. *Evolutionary Psychology*, *17*(2). <https://doi.org/10.1177/1474704919847450>

Filia, K., Teo, S. M., Brennan, N., Freeburn, T., Baker, D., Browne, V., Watson, A., Menssink, J., Prasad, A., Killackey, E., McGorry, P. D., Cotton, S. M., & Gao, C. X. (2025). Interrelationships between social exclusion, mental health and wellbeing in adolescents: insights from a national *Youth Survey*. *Epidemiology and Psychiatric Sciences,* *34*, e5. <https://doi.org/10.1017/S2045796024000878>

Finnish National Board on Research Integrity TENK (2023). Responsible Conduct of Research (RCR). Retrieved August 22, 2024 from: <https://tenk.fi/sites/default/files/2023-05/RI_Guidelines_2023.pdf>

Fisher, B. W., Viano, S., Curran, F. C., Pearman, F. A., & Gardella, J. H. (2018).  Students’ Feelings of Safety, Exposure to Violence and Victimization, and Authoritative School Climate.*American Journal of Criminal Justice: AJCJ, 43*(1), 6–25. <https://doi.org/10.1007/s12103-017-9406-6>

Fluck, J. (2017). Why Do Students Bully? An Analysis of Motives Behind Violence in Schools. *Youth & Society, 49*(5), 567–587. <https://doi.org/10.1177/0044118X14547876>

Forsberg, C., & Thornberg, R. (2016). The social ordering of belonging: Children’s perspectives on bullying. *International Journal of Educational Research, 78*, 13–23. <https://doi.org/10.1016/j.ijer.2016.05.008>

Forsberg, C., Wood, L., Smith, J., Varjas, K., Meyers, J., Jungert, T., & Thornberg, R. (2018). Students’ views of factors affecting their bystander behaviors in response to school bullying: a cross-collaborative conceptual qualitative analysis. *Research Papers in Education*, *33*(1), 127–142. <https://doi.org/10.1080/02671522.2016.1271001>

Fridh, M., Lindström, M., & Rosvall, M. (2019). Associations between self-injury and involvement in cyberbullying among mentally distressed adolescents in Scania, Sweden. *Scandinavian Journal of Public Health, 47*(2), 190–198. <https://doi.org/10.1177/1403494818779321>

Frisén, A., Hasselblad, T., & Holmqvist, K. (2012). What actually makes bullying stop? Reports from former victims. *Journal of Adolescence, 35*(4), 981–990. <https://doi.org/10.1016/j.adolescence.2012.02.001>

Gaffney, H., Ttofi, M. M., & Farrington, D. P. (2021a). Effectiveness of school‐based programs to reduce bullying perpetration and victimization: An updated systematic review and meta‐analysis. *Campbell Systematic Reviews, 17*(2), e1143. <https://doi.org/10.1002/cl2.1143>

Gaffney, H., Ttofi, M. M., & Farrington, D. P. (2021b). What works in anti-bullying programs? Analysis of effective intervention components. *Journal of School Psychology, 85*, 37–56. <https://doi.org/10.1016/j.jsp.2020.12.002>

Gan, X., Qin, K.-N., Xiang, G.-X., & Jin, X. (2023). The relationship between parental neglect and cyberbullying perpetration among Chinese adolescent: The sequential role of cyberbullying victimization and internet gaming disorder. *Frontiers in Public Health, 11*, 1128123. <https://doi.org/10.3389/fpubh.2023.1128123>

Garaigordobil, M., & Machimbarrena, J. M. (2019). Victimization and perpetration of bullying/cyberbullying: Connections with emotional and behavioral problems and childhood stress. *Psychosocial Intervention, 28*(2), 67–73. <https://doi.org/10.5093/pi2019a3>

Garaigordobil, M., & Martinez-Valderrey, V. (2015). Effects of cyberprogram 2.0 on 'face-to-face' bullying, cyberbullying, and empathy. *Psicothema, 27*(1), 45–51. <https://doi.org/10.7334/psicothema2014.78>

Garandeau, C. F., Laninga-Wijnen, L., & Salmivalli, C. (2022). Effects of the KiVa Anti-Bullying Program on Affective and Cognitive Empathy in Children and Adolescents. *Journal of Clinical Child & Adolescent Psychology, 51*(4), 515–529. <https://doi.org/10.1080/15374416.2020.1846541>

Garandeau, C. F., Lee, I. A., & Salmivalli, C. (2014). Differential effects of the KiVa anti-bullying program on popular and unpopular bullies. *Journal of Applied Developmental Psychology, 35*(1), 44–50. <https://doi.org/10.1016/j.appdev.2013.10.004>

Geng, J., Wang, Y., Wang, H., Wang, P., & Lei, L. (2022). Social Comparison Orientation and Cyberbullying Perpetration and Victimization: Roles of Envy on Social Networking Sites and Body Satisfaction. *Journal of Interpersonal Violence, 37*(17-18), NP16060-NP16083.
<https://doi.org/10.1177/08862605211023486>

Geoffroy, M.-C., Arseneault, L., Girard, A., Ouellet-Morin, I., & Power, C. (2023). Association of childhood bullying victimisation with suicide deaths: findings from a 50-year nationwide cohort study. *Psychological Medicine, 53*(9), 4152–4159. <https://doi.org/10.1017/S0033291722000836>

Graneheim, U. H., & Lundman, B. (2004). Qualitative content analysis in nursing research: concepts, procedures and measures to achieve trustworthiness. *Nurse Education Today 24*(2), 105–112. <https://doi.org/10.1016/j.nedt.2003.10.001>

Hammar, E., Bladh, M., & Agnafors, S. (2020). Mental health and experience of being bullied in 12-year-old children with overweight and obesity. *Acta Paediatrica, 109*(7), 1450–1457. <https://doi.org/10.1111/apa.15131>

Hellfeldt, K., López-Romero, L., & Andershed, H. (2020). Cyberbullying and Psychological Well-being in Young Adolescence: The Potential Protective Mediation Effects of Social Support from Family, Friends, and Teachers. *International Journal of Environmental Research and Public Health*, *17*(1), 45. <https://doi.org/10.3390/ijerph17010045>

Hellström, L., Persson, L., & Hagquist, C. (2015). Understanding and defining bullying – adolescents’ own views. *Archives of Public Health, 73*, 4. <https://doi.org/10.1186/2049-3258-73-4>

Hemphill, S. A., Tollit, M., & Herrenkohl, T. I. (2014). Protective Factors Against the Impact of School Bullying Perpetration and Victimization on Young Adult Externalizing and Internalizing Problems. *Journal of School Violence, 13*(1), 125–145. <https://doi.org/10.1080/15388220.2013.844072>

Hikmat, R., Yosep, I., Hernawaty, T., & Mardhiyah, A. (2024). A Scoping Review of Anti-Bullying Interventions: Reducing Traumatic Effect of Bullying Among Adolescents. *Journal of Multidisciplinary Healthcare, 17*, 289–304. <https://doi.org/10.2147/jmdh.s443841>

Holfeld, B., & Baitz, R. (2020). The Mediating and Moderating Effects of Social Support and School Climate on the Association between Cyber Victimization and Internalizing Symptoms. *Journal of Youth and Adolescence, 49*(11), 2214–2228. <https://doi.org/10.1007/s10964-020-01292-0>

Holt, M. K., Vivolo-Kantor, A. M., Polanin, J. R., Holland, K. M., DeGue, S., Matjasko, J. L., Wolfe, M., & Reid, G. (2015). Bullying and Suicidal Ideation and Behaviors: A Meta-Analysis. *Pediatrics, 135*(2), e496–e509. <https://doi.org/10.1542/peds.2014-1864>

Hopkins, L., Taylor, L., Bowen, E., & Wood, C. (2013). A qualitative study investigating adolescents' understanding of aggression, bullying and violence. [*Children and Youth Services Review*](https://www-sciencedirect-com.ezproxy.vasa.abo.fi/journal/children-and-youth-services-review)*, 35*(4), 685–693. <https://doi.org/10.1016/j.childyouth.2013.01.012>

Huitsing, G., Lodder, G. M. A., Browne, W. J., Oldenburg, B., Van der Ploeg, R., & Veenstra, R. (2020). A Large-Scale Replication of the Effectiveness of the KiVa Antibullying Program: a Randomized Controlled Trial in the Netherlands. *Prevention Science, 21*(5), 627–638. <https://doi.org/10.1007/s11121-020-01116-4>

Huitsing, G., Snijders, T. A. B., Van Duijn, M. A. J., & Veenstra, R. (2014). Victims, bullies, and their defenders: A longitudinal study of the coevolution of positive and negative networks. *Development and Psychopathology, 26*(3), 645–659. <https://doi.org/10.1017/S0954579414000297>

Islam, M. I., Yunus, F. M., Salam, S. S., Kabir, E., & Khanam, R. (2022). Mental health services use among adolescent bullying victims in Australia: Results from a nationwide survey. *Children and Youth Services Review, 132*, 106312. <https://doi.org/10.1016/j.childyouth.2021.106312>

Jaskulska, S., Jankowiak, B., Pérez-Martínez, V., Pyżalski, J., Sanz-Barbero, B., Bowes, N., Claire, K. D., Neves, S., Topa, J., Silva, E., Mocanu, V., Gena Dascalu, C., & Vives-Cases, C. (2022). Bullying and Cyberbullying Victimization and Associated Factors among Adolescents in Six European Countries. *Sustainability, 14*(21), 14063. <https://doi.org/10.3390/su142114063>

Johansson, A., Huhtamäki, A., Sainio, M., Kaljonen, A., Boivin, M., & Salmivalli, C. (2022). Heritability of Bullying and Victimization in Children and Adolescents: Moderation by the KiVa Antibullying Program. *Journal of Clinical Child & Adolescent Psychology, 51*(4), 505–514. <https://doi.org/10.1080/15374416.2020.1731820>

Karatuna, I. (2015). Targets’ coping with workplace bullying: a qualitative study*. Qualitative Research in Organizations and Management, 10*(1), 21–37. <https://doi.org/10.1108/QROM-09-2013-1176>

Klomek, A. B., Marrocco, F., Kleinman, M., Schonfeld, I. S., Gould, M. S. (2007). Bullying, Depression, and Suicidality in Adolescents. *Journal of the American Academy of Child & Adolescent Psychiatry*. *46*(1): 40–49. <https://doi.org/10.1097/01.chi.0000242237.84925.18>

Kowalski, R. M., & Limber, S. P. (2013). Psychological, physical, and academic correlates of cyberbullying and traditional bullying. *Journal of Adolescent Health, 53*(1), S13-S20. <https://doi.org/10.1016/j.jadohealth.2012.09.018>

Koyanagi, A., Oh, H., Carvalho, A. F., Smith, L., Haro, J. M., Vancampfort, D., Stubbs, B., & DeVylder, J. E. (2019). Bullying Victimization and Suicide Attempt Among Adolescents Aged 12–15 Years From 48 Countries. *Journal of the American Academy of Child & Adolescent Psychiatry, 58*(9), 907–918. <https://doi.org/10.1016/j.jaac.2018.10.018>

Laitinen, K., Haanpää, S., Francke, L., & Lahtinen, M. (2020). *Kiusaamisen vastainen työ kouluissa ja oppilaitoksissa* (Oppaat ja käsikirjat 2020:3a). Opetushallitus. <https://www.oph.fi/fi/tilastot-ja-julkaisut/julkaisut/kiusaamisen-vastainen-tyo-kouluissa-ja-oppilaitoksissa>

Lambe, L. J., Hudson, C. C., Craig, W. M., & Pepler, D. J. (2017). Does defending come with a cost? Examining the psychosocial correlates of defending behaviour among bystanders of bullying in a Canadian sample. *Child Abuse & Neglect, 65*, 112–123. <https://doi.org/10.1016/j.chiabu.2017.01.012>

Laninga-Wijnen, L., van den Berg, Y. H. M., Garandeau, C. F., Mulder, S., & de Castro, B. O. (2023). Does being defended relate to decreases in victimization and improved psychosocial adjustment among victims? *Journal of Educational Psychology, 115*(2), 363–377. <https://doi.org/10.1037/edu0000712>

Lechner, V., Crăciun, I. C., & Scheithauer, H. (2023). Barriers, resources, and attitudes towards (cyber-)bullying prevention/intervention in schools from the perspective of school staff: Results from focus group discussions. *Teaching and Teacher Education, 135*, 104358. <https://doi.org/10.1016/j.tate.2023.104358>

Lidberg, J., Berne, S., & Frisén, A. (2024). From childhood bullying victimization to resilience in emerging adulthood. *Scandinavian Journal of Psychology, 65*(3), 521–532. <https://doi.org/10.1111/sjop.12999>

Lin, Y.-C., Latner, J. D., Fung, X. C. C., & Lin, C.-Y. (2018). Poor Health and Experiences of Being Bullied in Adolescents: Self-Perceived Overweight and Frustration with Appearance Matter. *Obesity, 26*(2), 397–404. <https://doi.org/10.1002/oby.22041>

Låftman, S. B., Grigorian, K., Lundin, A., Östberg, V., & Raninen, J. (2024). Bullying experiences before and after the transition from lower to upper secondary school: associations with subsequent mental health in a Swedish cohort. *BMC Public Health, 24*, 27. <https://doi.org/10.1186/s12889-023-17443-4>

Ma, T.-L., & Chen, W.-T. (2019). The benefits of being defended: Perceived bystander participant roles and victims’ emotional and psychosocial adjustment. *Journal of School Violence*, *18*(1), 77–91. <https://doi.org/10.1080/15388220.2017.1387132>

Madsen, K. R., Damsgaard, M. T., Petersen, K., Qualter, P., & Holstein, B. E. (2024). Bullying at School, Cyberbullying, and Loneliness: National Representative Study of Adolescents in Denmark. *International Journal of Environmental Research and Public Health*, *21*(4), 414. <https://doi.org/10.3390/ijerph21040414>

Malamut, S. T., Trach, J., Garandeau, C. F., & Salmivalli, C. (2023). Does defending victimized peers put youth at risk of being victimized? *Child Development, 94*(2), 380–394. <https://doi.org/10.1111/cdev.13866>

Masoumi, D., Bourbour, M., Edling, S., Gill, P., & Francia, G. (2024). School Staff Strategies for Identifying, Dealing with and Preventing Cyberbullying Among Swedish Primary School Pupils. *Computers in the Schools*, 1–22. <https://doi.org/10.1080/07380569.2024.2416426>

Mazzone, A., Thornberg, R., Stefanelli, S., Cadei, L., & Caravita, S. C. S. (2018). “Judging by the cover”: A grounded theory study of bullying towards same-country and immigrant peers. *Children and Youth Services Review, 91*, 403–412. <https://doi.org/10.1016/j.childyouth.2018.06.029>

McGrath, M. Z. (2006). *School Bullying: Tools for Avoiding Harm and Liability*. Thousand Oaks, Calif: Corwin Press. p. 21.

Mehari, K. R., Moore, W., Waasdorp, T. E., Varney, O., Berg, K., & Leff, S. S. (2018). Cyberbullying prevention: Insight and recommendations from youths, parents, and paediatricians. *Child: Care, Health and Development, 44*(4), 616–622. <https://doi.org/10.1111/cch.12569>

Merrill, R. M., & Hanson, C. L. (2016). Risk and protective factors associated with being bullied on school property compared with cyberbullied. *BMC Public Health, 16*, 145. <https://doi.org/10.1186/s12889-016-2833-3>

Mischel, J., & Kitsantas, A. (2020). Middle school students’ perceptions of school climate, bullying prevalence, and social support and coping. *Social Psychology of Education, 23*(1), 51–72 <https://doi.org/10.1007/s11218-019-09522-5>

Munawaroh, E., Sutoyo, A., Arinata, F. S., Karomah, A., Damayanti, S., & Nitami, L. Z. (2024). The Protective Factors of Bullying Victimization Experiences on Mental Health among Adolescents: A Decade of Systematic Review. *KONSELOR, 13*(2), 72–82 <https://doi.org/10.24036/0202413262-0-86>

Murphy, T. P., Laible, D., & Augustine, M. (2017). The Influences of Parent and Peer Attachment on Bullying. *Journal of Child and Family Studies, 26*(5), 1388–1397. <https://doi.org/10.1007/s10826-017-0663-2>

Nocentini, A., & Menesini, E. (2016). KiVa Anti-Bullying Program in Italy: Evidence of Effectiveness in a Randomized Control Trial. *Prevention Science, 17*(8), 1012–1023. <https://doi.org/10.1007/s11121-016-0690-z>

Noret, N., Hunter, S. C., & Rasmussen, S. (2020). The Role of Perceived Social Support in the Relationship Between Being Bullied and Mental Health Difficulties in Adolescents. *School Mental Health, 12*(1), 156–168. <https://doi.org/10.1007/s12310-019-09339-9>

Olweus, D. (1994). Bullying at School: Basic Facts and Effects of a School Based Intervention Program. *Journal of Child Psychology and Psychiatry, 35*(7), 1171–1190. <https://doi.org/10.1111/j.1469-7610.1994.tb01229.x>

Opetushallitus. (2025). *Kiusaamisen ehkäiseminen ja turvallisuutta edistävät toimintamallit.* <https://www.oph.fi/fi/koulutus-ja-tutkinnot/kiusaamisen-ehkaiseminen-ja-turvallisuutta-edistavat-toimintamallit> (Retrieved 06.02.2025)

Palade, T., & Pascal, E. (2023). Reducing Bullying through Empathy Training: The Effect of Teacher’s Passive Presence. *Behavioral Sciences, 13*(3), 216. <https://doi.org/10.3390/bs13030216>

Parisse, C., Marini, M., Prislei, L., Scarci, F., Cecalupo, A., & Livi, S. (2024). Perceived class cohesion as a protection strategy against cyber-bullying in vulnerable students: A study of secondary school students. *Journal of Community & Applied Social Psychology, 34*(5), e2876. <https://doi.org/10.1002/casp.2876>

Peter, I.-K., & Petermann, F. (2018). Cyberbullying: A concept analysis of defining attributes and additional influencing factors. *Computers in Human Behavior, 86*, 350–366. <https://doi.org/10.1016/j.chb.2018.05.013>

Prince, T., Mulgrew, K. E., Driver, C., Mills, L., Loza, J., & Hermens, D. F. (2024). Appearance-related cyberbullying and its association with the desire to alter physical appearance among adolescent females. *Journal of Eating Disorders, 12*, 125. <https://doi.org/10.1186/s40337-024-01083-z>

Ramirez, M. R., Seedorff, J., Cavanaugh, J. E., Ryan, A., Xiong, B. N., & Hatzenbuehler, M. L. (2024). Does Implementation Matter? Associations Between Implementation of Maine's Anti- Bullying Law and Bullying Victimization Among High School Youth. *Journal of Adolescent Health, 74*(1), 161–168. <https://doi.org/10.1016/j.jadohealth.2023.08.011>

Reason, L., Boyd, M., & Reason, C. (2016). Cyberbullying in Rural Communities: Origin and Processing through the Lens of Older Adolescents. *The Qualitative Report, 21*(12), 2331–2348.
<https://doi.org/10.46743/2160-3715/2016.2554>

Sainio, M., Herkama, S., Turunen, T., Rönkkö, M., Kontio, M., Poskiparta, E., & Salmivalli, C. (2020). Sustainable antibullying program implementation: School profiles and predictors. *Scandinavian Journal of Psychology, 61*(1), 132–142. <https://doi.org/10.1111/sjop.12487>

Salmivalli, C., Laninga-Wijnen, L., Malamut, S. T., & Garandeau, C. F. (2021). Bullying Prevention in Adolescence: Solutions and New Challenges from the Past Decade. *Journal of Research on Adolescence, 31*(4), 1023–1046. <https://doi.org/10.1111/jora.12688>

Schacter, H. L., & Juvonen, J. (2020). When Do Friendships Help Versus Hurt? Perceived Best Friend Victimization and Support as Moderators of Peer Victimization-Related Distress. *The Journal of Early Adolescence, 40*(6), 804–827. <https://doi.org/10.1177/0272431619874402>

Stegmair, J., & Prybutok, V. (2024). Cyberbullying and Resilience: Lessons Learned from a Survey. *Societies*, *14*(8), 137. <https://doi.org/10.3390/soc14080137>

Strindberg, J. (2023). “I’m Often Alone”: An Ethnographic Study of School Loneliness and Bullying in a Swedish Elementary School. *Child & Youth Care Forum, 52*(4), 875–892. <https://doi.org/10.1007/s10566-022-09715-8>

Strindberg, J., Horton, P., & Thornberg, R. (2020). The fear of being singled out: pupils’ perspectives on victimisation and bystanding in bullying situations. *British Journal of Sociology of Education, 41*(7), 942–957. <https://doi.org/10.1080/01425692.2020.1789846>

Tang, J. J., Yu, Y., Wilcox, H. C., Kang, C., Wang, K., Wang, C., Wu, Y., & Chen, R. (2020). Global risks of suicidal behaviours and being bullied and their association in adolescents: School-based health survey in 83 countries. *eClinicalMedicine, 19*, 100253. <https://doi.org/10.1016/j.eclinm.2019.100253>

Thornberg, R. (2015a). Distressed Bullies, Social Positioning and Odd Victims: Young People’s Explanations of Bullying. *Children & Society, 29*(1), 15–25. <https://doi.org/10.1111/chso.12015>

Thornberg, R. (2015b). School Bullying as a Collective Action: Stigma Processes and Identity Struggling. *Children & Society, 29*(4), 310–320. <http://dx.doi.org/10.1111/chso.12058>

Thornberg, R. (2018). School bullying and fitting into the peer landscape: a grounded theory field study. *British Journal of Sociology of Education, 39*(1), 144–158. <https://doi.org/10.1080/01425692.2017.1330680>

Thornberg, R., Landgren, L., & Wiman, E. (2018). ‘It Depends’: A qualitative study on how adolescent students explain bystander intervention and non-intervention in bullying situations. *School Psychology International, 39*(4), 400–415. <https://doi.org/10.1177/0143034318779225>

Thornberg, R., Tenenbaum, L., Varjas, K., Meyers, J., Jungert, T., & Vanegas, G. (2012). Bystander Motivation in Bullying Incidents: To Intervene or Not to Intervene? *Western Journal of Emergency Medicine 13*(3), 247–252. <https://doi.org/10.5811/westjem.2012.3.11792>

Tong, A., Sainsbury, P., & Craig, J. (2007). Consolidated criteria for reporting qualitative research (COREQ): A 32-item checklist for interviews and focus groups. *International Journal of Quality in Health Care, 19*(6), 349–357. <http://intqhc.oxfordjournals.org/content/19/6/349.long>

Turanovic, J. J., Siennick, S. E., & Lloyd, K. M. (2023). Consequences of Victimization on Perceived Friend Support during Adolescence. *Journal of Youth and Adolescence, 52*(3), 519–532. <https://doi.org/10.1007/s10964-022-01706-1>

Underwood, M. K. (2003). *Social Aggression among Girls. (Guilford Series On Social And Emotional Development)*. New York: The Guilford Press.

van der Ploeg, R., Steglich, C., & Veenstra, R. (2016). The support group approach in the Dutch KiVa anti-bullying programme: effects on victimisation, defending and well-being at school. *Educational Research*, *58*(3), 221–236.  [https://doi.org/10.1080/00131881.2016.1184949](https://doi-org.ezproxy.vasa.abo.fi/10.1080/00131881.2016.1184949)

van Geel, M., Vedder, P., & Tanilon, J. (2014). Are overweight and obese youths more often bullied by their peers? A meta-analysis on the relation between weight status and bullying. *International Journal of Obesity, 38*(10), 1263–1267. <https://doi.org/10.1038/ijo.2014.117>

Varela, J. J., Muñoz-Najar Pacheco, A., Chuecas, M. J., Rodríguez-Rivas, M. E., Guzmán, P., & Mattar Yunes, M. A. (2022). Life Satisfaction, Bullying, and Feeling Safe as a Protective Factor for Chilean and Brasilian Adolescents. *Child Indicators Research, 15*(2), 579–598. <https://doi.org/10.1007/s12187-021-09872-7>

Veenstra, R., Lindenberg, S., Huitsing, G., Sainio, M., & Salmivalli, C. (2014). The role of teachers in bullying: The relation between antibullying attitudes, efficacy, and efforts to reduce bullying. *Journal of Educational Psychology, 106*(4), 1135–1143. <http://dx.doi.org/10.1037/a0036110>

Villanueva-Moya, L., Herrera, M. C., Sánchez-Hernández, M. D., & Expósito, F. (2023). #Instacomparison: Social Comparison and Envy as Correlates of Exposure to Instagram and Cyberbullying Perpetration. *Psychological Reports*, *126*(3), 1284–1304. <https://doi.org/10.1177/00332941211067390>

Virrankari, L., Leemann, L., & Kivimäki, H. (2020). *Osallisuuden kokemus ja koulukiusaaminen: Kouluterveyskyselyn 2019 tuloksia* (TUTKIMUKSESTA TIIVIISTI 35/2020). THL. <https://urn.fi/URN:ISBN:978-952-343-599-5>

Volk, A. A., Dane, A. V., & Marini, Z. A. (2014). What is bullying? A theoretical redefinition. *Developmental Review, 34*(4), 327–343. <https://doi.org/10.1016/j.dr.2014.09.001>

Wang, C.-W., Musumari, P. M., Techasrivichien, T., Suguimoto, S. P., Chan, C.-C., Ono-Kihara, M., Kihara, M., & Nakayama, T. (2019). “I felt angry, but I couldn’t do anything about it”: a qualitative study of cyberbullying among Taiwanese high school students. *BMC Public Health, 19*, 654. <https://doi.org/10.1186/s12889-019-7005-9>

Wang, X., Yang, L., Gao, L., Yang, J., Lei, L., & Wang, C. (2017). Childhood maltreatment and Chinese adolescents' bullying and defending: The mediating role of moral disengagement. *Child Abuse & Neglect, 69*, 134–144. <https://doi.org/10.1016/j.chiabu.2017.04.016>

Wang, Z., & Jiang. S. (2022). Influence of parental neglect on cyberbullying perpetration: Moderated mediation model of smartphone addiction and self-regulation. *Health and Social Care in the Community, 30*(6), 2372–2382. <https://doi.org/10.1111/hsc.13787>

White, R. L., Babic, M. J., Parker, P. D., Lubans, D. R., Astell-Burt, T., & Lonsdale, C. (2017). Domain-Specific Physical Activity and Mental Health: A Meta-analysis. *American Journal of Preventive Medicine, 52*(5), 653–666. <https://doi.org/10.1016/j.amepre.2016.12.008>

Wilkinson, I. G. (2017). Bullying in Canada in the 21st Century: The Moral Obligations of Parents, Teachers, Schools and Governments. *The International Journal of Children's Rights, 25*(1), 231–260. <https://doi.org/10.1163/15718182-02501010>

Wilson, D. S., Near, D., & Miller, R. R. (1996). Machiavellianism: A synthesis of the evolutionary and psychological literatures. *Psychological Bulletin, 119*(2), 285–299. <https://doi.org/10.1037/0033-2909.119.2.285>

Wójcik, M. (2018). The parallel culture of bullying in Polish secondary schools: A grounded theory study. *Journal of Adolescence, 69*(1), 72–79. <https://doi.org/10.1016/j.adolescence.2018.09.005>

Wójcik, M., & Rzeńca, K. (2021). Disclosing or Hiding Bullying Victimization: A Grounded Theory Study From Former Victims’ Point of View. *School Mental Health, 13*(4), 808–818. <https://doi.org/10.1007/s12310-021-09447-5>

Wood, L., Smith, J., Varjas, K., & Meyers, J. (2017). School personnel social support and nonsupport for bystanders of bullying: Exploring student perspectives. *Journal of School Psychology, 61*, 1–17. <https://doi.org/10.1016/j.jsp.2016.12.003>

World Health Organization. (10 oktober 2024a). *Mental health of adolescents*. <https://www.who.int/news-room/fact-sheets/detail/adolescent-mental-health> (Hämtad 22.01.2025)

World Health Organization. (27 mars 2024b). *One in six school-aged children experiences cyberbullying, finds new WHO/Europe study*. <https://www.who.int/europe/news/item/27-03-2024-one-in-six-school-aged-children-experiences-cyberbullying--finds-new-who-europe-study> (Hämtad 16.11.2024)

Yablon, Y. B. (2020). Combining Teaching and Counseling Roles: Implications for Students’ Willingness to Seek Help for Bullying. *International Journal for the Advancement of Counselling, 42*(4), 382–392. <https://doi.org/10.1007/s10447-020-09407-9>

Yang, L., Xiong, Y., Gao, T., Li, S., & Ren, P. (2023). A person-centered approach to resilience against bullying victimization in adolescence: Predictions from teacher support and peer support. *Journal of Affective Disorders, 341*, 154–161. <https://doi.org/10.1016/j.jad.2023.08.089>

Ybarra, M. L., Espelage, D. L., Valido, A., Hong, J. S., & Prescott, T. L. (2019). Perceptions of middle school youth about school bullying. *Journal of Adolescence, 75*(1), 175–187. <https://doi.org/10.1016/j.adolescence.2018.10.008>

Yeager, D. S., Fong, C. J., Lee, H. Y., & Espelage, D. L. (2015). Declines in efficacy of anti-bullying programs among older adolescents: Theory and a three-level meta-analysis. *Journal of Applied Developmental Psychology, 37*, 36–51. <https://doi.org/10.1016/j.appdev.2014.11.005>

Yu, S., & Zhao, X. (2021). The negative impact of bullying victimization on academic literacy and social integration: Evidence from 51 countries in PISA. *Social Sciences & Humanities Open, 4*(1), 100151. <https://doi.org/10.1016/j.ssaho.2021.100151>

Zapata-López, J. S., Tonguino-Rosero, S., & Méndez, F. (2024). Sedentary Behavior and School Bullying in Adolescents: An Analysis Based on a National Survey in Colombia. *Journal of School Health, 94*(8), 727–735. <https://doi.org/10.1111/josh.13397>

Zhu, Y. (2023). ‘Big brothers and sisters have my back’: Benefits and risks of befriending older peers as a strategy to deal with school bullying. *Child Abuse Review, 32*(3), e2827. <https://doi.org/10.1002/car.2827>

**Tables**

Table 1. Example of the analysis

| **Meaning units** | **Condensation** | **Code** | **Subcategory** | **Main category** |
| --- | --- | --- | --- | --- |
| *Psychological bullying... Well, I’ve been overweight so I’ve been bullied because of it* | Bullied because of being overweight | Overweight | *To deviate from the norm or personal difficulties* | **Causes of bullying and intervention and mitigating aspects** |

Table 2. Overview of the results

| Main category | Subcategory |
| --- | --- |
| Causes of bullying and intervention and mitigating aspects | *To deviate from the norm or personal difficulties*  *Intervention by schools and adults*  *Obstacles to intervention and action* |
| Protection against the harmful effects of bullying and loneliness | *Close and secure relationships as support*  *Change of environment, school transitions and own characteristics and actions* |
| Prevention and promotion of well-being as solutions | *Safe meeting places for inclusion and collaboration as well as professional support*  *Community, security and openness*  *Upbringing and dissemination of knowledge*  *Engagement and intervention* |
